# Waist-hip Ratio (WHR), a Better Predictor for Prostate Cancer than Body Mass Index (BMI): Results from a Chinese Hospital-based Biopsy Cohort

**DOI:** 10.1038/srep43551

**Published:** 2017-03-08

**Authors:** Bo Tang, Cheng-Tao Han, Gui-Ming Zhang, Cui-Zhu Zhang, Wei-Yi Yang, Ying Shen, Adriana C. Vidal, Stephen J. Freedland, Yao Zhu, Ding-Wei Ye

**Affiliations:** 1 Department of Urology, Fudan University Shanghai Cancer Center, Shanghai, China; 2 Department of Oncology, Shanghai Medical Colleague, Fudan University, Shanghai, China; 3 Department of Surgery, Center for Integrated Research on Cancer and Lifestyle, Samuel Oschin Comprehensive Cancer Institute, Cedars Sinai Medical Center, Los Angeles, CA

## Abstract

To investigate whether waist-hip ratio (WHR) is a better predictor of prostate cancer (PCa) incidence than body mass index (BMI) in Chinese men. Of consecutive patients who underwent prostate biopsies in one tertiary center between 2013 and 2015, we examined data on 1018 with PSA ≤20 ng/ml. Clinical data and biopsy outcomes were collected. Logistic regression was used to evaluate the associations between BMI, WHR and PCa incidence. Area under the ROC (AUC) was used to evaluate the accuracy of different prognostic models. A total of 255 men and 103 men were diagnosed with PCa and high grade PCa (HGPCa, Gleason score ≥8). WHR was an independent risk factor for both PCa (OR = 1.07 95%Cl 1.03–1.11) and HGPCa (OR = 1.14 95%Cl 1.09–1.19) detection, while BMI had no relationship with either PCa or HGPCa detection. Adding WHR to a multivariable model increased the AUC for detecting HGPCa from 0.66 (95%Cl 0.60–0.72) to 0.71 (95%Cl 0.65–0.76). In this Chinese cohort, WHR was significantly predictive of PCa and HGPCa. Adding WHR to a multivariable model increased the diagnostic accuracy for detecting HGPCa. If confirmed, including WHR measurement may improve PCa and HGPCa detection.

Prostate cancer (PCa) is one of the most common cancers in men around the world, second after lung, bronchus and trachea cancers^1^. In China, PCa incidence and mortality rates have been rapidly increasing, with an estimate of 60,300 new cases and 26,600 deaths in 2015 only^2^.

Obesity is associated with the development of several cancers^3^. However, worldwide, studies on the association between body mass index (BMI) and PCa incidence are still controversial^4^,^5^,^6^. Indeed, among Asian men, several studies reported that a higher BMI had either positive or negative effects on the risk of PCa and high-grade PCa (HGPCa)^7^,^8^,^9^. One recent study considered the fact that BMI values in Asians are lower than in Western counterparts and thus used a BMI cut-off of 25 kg/m^2^ to define obesity. In doing so, they found that higher BMI was linked with increased risk of PCa at biopsy, however no significant association was seen between obesity and HGPCa^8^. A recent report also found that Asian men with a BMI ≥25 kg/m^2^ were at a higher risk of having PCa at initial biopsy^9^, confirming that indeed obesity may be associated with higher risk of total PCa, although the associations between obesity and PCa grade among Asian men are still unclear.

One reason that could explain those discrepancies is that although BMI is the most commonly used measurement of obesity likely due to its ease of use, other measurements can reflect differences in body fat distribution and represent different types of obesity^10^, which may be more strongly linked with PCa and HGPCa. For example, waist-hip ratio (WHR), measured as waist circumference divided by hip circumference, reflects abdominal fat accumulation, and has been shown to be associated with PCa in white and black men^11^,^12^,^13^,^14^, and also related to PCa grade^11^,^12^,^13^. But few studies to date included Asian men^15^,^16^. In this study, we evaluated whether WHR and BMI predict PCa detection and specifically HGPCa among men undergoing prostate biopsies in China. We hypothesized that while both BMI and WHR would predict PCa risk and HGPCa, associations would be stronger for WHR.

## Results

### Study participants’ characteristics

Tables 1 and 2 shows patients’ characteristics, stratified by biopsy findings. A total of 255 out of 1,018 men (25%) were diagnosed with PCa. Patients with PCa were older, had higher PSA levels, lower %fPSA, smaller prostates, and were more likely to have an abnormal DRE, abnormal TRUS or MRI, and had higher WHR (all p < 0.05). Cigarette use, alcohol use, hypertension, and diabetes mellitus were not associated with cancer detection on biopsy (all p > 0.05). Only a modest percent of men with PCa had Gleason score 6 (22%), with most having Gleason score 7 (38%) or Gleason 8–10 (40%) (Table 1). Patients with PCa had no significant difference in BMI compared to men without cancer (p = 0.962). Similarly, there was no difference in BMI between men with HGPCa, low grade PCa, and non-cancer (p = 0.924) (Table 2). Supplementary Fig 1a and b display the distribution of BMI and WHR in the overall study population.

Patients’ BMI and WHR were both normally distributed. According to Asian criteria^17^, 39.3% patients had under or normal weights (BMI < 23 kg/m^2^), 32.4% were overweight (23 ≤ BMI < 25 kg/m^2^), and 28.3% were obese (BMI ≥25 kg/m^2^). A total of 529 (52.0%) men had abdominal obesity, defined as WHR above 0.90 for males, according to the WHO expert consultation in 2008^18^.

### BMI, WHR and PCa & HGPCa detection

On multivariable analysis, when treated as a continuous variable, there was no significant relationship between BMI and PCa detection (p = 0.473) and HGPCa detection (p = 0.932) (Table 3). Similarly, when treated as a categorical variable, BMI still had no association with either PCa or HGPCa detection (Table 4).

Patients with PCa had significantly higher WHR compared to men without cancer (p = 0.002). Similarly, men with HGPCa had higher WHR than men with low grade PCa and men without PCa (p < 0.001) (Table 2). On multivariable analysis, higher WHR was statistically significantly correlated with higher risk of PCa (OR = 1.07, 95%Cl 1.03–1.11, P = 0.001) and HGPCa (OR = 1.14, 95%Cl 1.09–1.19, P < 0.001) detection (Table 3). For every 0.01 increase of WHR, the probability of men in our study having PCa and HGPCa increased 7% and 14%, respectively.

### Predictive accuracy of PSA and multi-variable model

**Figure 1:**
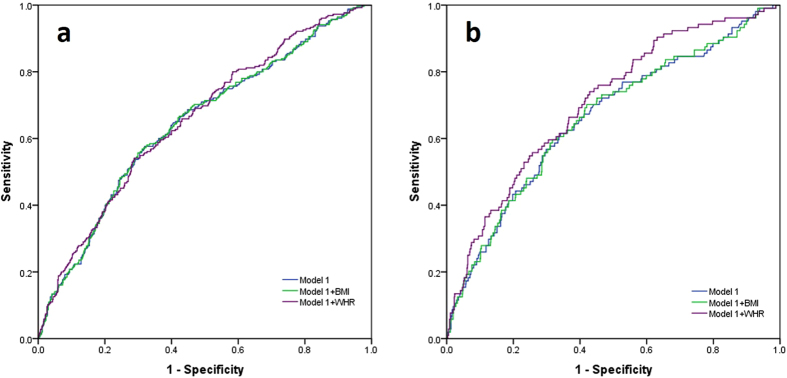
(**a**). ROC curves for different models in predicting PCa detection. (**b**). ROC curves for different models in predicting HGPCa detection.

For our study population, we constructed a multivariable model to predict PCa risk (Table 5, Fig. 1). The model 1 based on PSA, DRE results, and imaging (TURS or MRI) findings, which represented the various indications for biopsy we used in our population. The AUC of this model in predicting PCa and HGPCa was 0.645 and 0.660, respectively. Addition of WHR to this model significantly increased AUC in HGPCa detection (AUC = 0.706 95%Cl 0.653–0.758, p = 0.025). Adding WHR to the model for detecting PCa also improved the AUC (from 0.645 to 0.655), though the increase was not statistically significant (p = 0.245). In contrast, adding BMI to the model had almost no changes in diagnostic accuracy for either PCa or HGPCa detection (Table 5).

## Discussion

This is the first study to analyze the relationship between WHR and PCa detection in a Chinese hospital-based biopsy cohort. We found that WHR significantly improved prediction of PCa and HGPCa in Chinese men while BMI did not. Specifically, for HGPCa detection, with every 0.01 increase of WHR, the probability of men having HGPCa increased 13%. Moreover, when WHR was added to a multivariable model, the diagnostic accuracy for detecting HGPCa was improved. If confirmed in future studies, WHR may be a useful adjunct to detect men scheduled for a prostate biopsy who are at the highest risk of HGPCa.

While the effect of BMI on PCa incidence has been well studied, results continue to be mixed. Indeed, some studies found that BMI is a risk factor for PCa^6^,^19^, others reported no association between BMI and PCa^4^, and yet others found BMI had a protective effect on PCa diagnosis^5^. Little evidence of a substantial effect of genetically elevated height or BMI on prostate cancer risk was found^20^. The consensus that has been reached recently on this issue among white men was that BMI was associated with decreased risk of low-grade PCa but with increased risk of high-grade PCa^21^,^22^,^23^. However, there are limitations with the use of BMI in that it can be influenced by factors other than obesity. For example, high muscle mass can create a high BMI in the absence of obesity. Alternatively, men could have sarcopenic obesity wherein BMI is normal, though the men have metabolic effects from obesity. Even if BMI does correlate with excess adiposity, the anatomic location of the excess fat can vary. Specifically, adipose tissues appear in multiple locations throughout the body, the distribution of which has clinical importance with central adiposity, especially visceral obesity, being more strongly linked with adverse metabolic effects^24^. However, BMI does not reflect this point. Alternatively, WHR is an economical and practical body measurement that better reflects visceral obesity in clinical practice.

Several studies have examined the association between WHR and PCa detection risk. In an American retrospective study of 500 men^11^, WHR was significantly associated with HGPCa only among men without prostate enlargement, but not associated with total PCa detection. In a larger population-based case-control study in Canada^12^ with over 1,900 cases and over 1,900 controls, a WHR > 1.0 was associated with increased risk of HGPCa (OR = 1.20 95%Cl 1.01–1.43) but no association was found between WHR and total PCa detection. The two studies above were multiracial, but white men accounted for more than 85% subjects in both studies. Data from other geographic regions showed different results. In three case-control studies from North India^15^, Jamaica^13^ and Barbados^14^, WHR was significantly higher in both PCa and HGPCa patients. A study^16^ in a Chinese population showed that high levels of WHR were related to an increased risk of PCa detection, with men in the highest quartile (WHR > 0.92) having almost a 3-fold risk (OR = 2.71 95%CI 1.66–4.41 P < 0.001) compared with men in the lowest quartile (WHR < 0.86), similar to our results. PSA and Gleason score, two of the most important indicators in PCa diagnose and prognosis, were not mentioned in the research.

Given the above data, there seems to be some consensus that WHR is associated with HGPCa, but the relationship between WHR and total PCa detection is controversial in different races. In our study, WHR was associated with both PCa and HGPCa detection, but the link between WHR and HGPCa detection was more remarkable. Some mechanisms may help explain the positive association between WHR and PCa progression. Insulin-like growth factor (IGF)-1 has been widely involved in tumorigenesis^25^. Elevated circulating IGF-1 has been linked to increased PCa incidence^26^. Meanwhile, one study showed that people with lower WHR tend to have a low serum IGF-1^27^. Several adipokines (leptin and adiponectin), which are associated with WHR, have been postulated to modulate PCa development and growth. In human androgen-independent PC-3 and DU145 PCa cell lines, leptin promotes tumor growth^28^. A few studies found that leptin had consistent change trend with WHR during the follow-up of many diseases^29^,^30^. In contrast to the above, adiponectin often plays an antitumor role. Adiponectin levels were found to be reduced significantly in metastatic PCa patients versus those with organ-confined disease^31^, but had no association with overall PCa risk in a prospective study^32^. At the same time, a significant negative correlation between serum concentration of adiponectin and WHR was found^33^.

Several strengths in our study should be emphasized. As for data selection, we only chose PSA ≤ 20 ng/ml men in our study analysis for two reasons. First, PSA ≤ 20 ng/ml, known as the gray zone of PCa diagnose in China, is the area wherein there is greatest concern for over-diagnosis and overtreatment^34^. The harms of over-diagnosis in PSA testing may cause unnecessary infections and other complications^35^. Therefore, patients with PSA ≤ 20 ng/ml require better screening tools to detect aggressive PCa early while avoiding unnecessary biopsies in those who are low risk. Second, advanced PCa patients tend to have high level PSA and cancer cachexia would cause changes of BMI and WHR which may have caused bias in our data analyses. As for data analyses, we used multivariable analysis to adjust for key covariates. In addition, we analyzed WHR as a continuous variable to avoid use of arbitrary cutoff values. Different studies have used different cutoff values of WHR which makes comparing results challenging, particular as it relates to analyses from different races. For instance, people in developed countries tend to have more abdominal fat. In the study in North-America^12^, 36% of the men had a WHR > 1.0. In our study, only 9 men (0.9%) had WHR > 1.0. Finally, it is important to note that no study to date has tested whether adding WHR to a multivariable model improves detection accuracy. In this study, we found that adding WHR to a multivariable model significantly increased the AUC of HGPCa detection.

Nonetheless, limitations should be acknowledged. First, as one of the tertiary health institutes and one of only a few dedicated centers in mainland China, patients from all over the country come to our department. However, our study is limited in that it is a retrospective study from only one institute. Even if our conclusion applied broadly to Chinese and the entire Asian population, it may not apply to white or black men. Second, as a retrospective study, we measured patients’ body data accurately before biopsy, but we can’t assess patients’ WHR or BMI over the past ten or twenty years, which may be in a constantly changing process in such a long period. Since we used the questionnaire, recall bias surely existed. Patients may not accurately recall living habits during past decades. Third, while our study demonstrated a significant association, further work is needed to understand the biological basis for this finding. Nevertheless, the results of this study provided additional information toward more precise PCa diagnoses among men with increased abdominal fat.

## Conclusion

In this Chinese hospital-based prostate biopsy cohort, we showed WHR significantly improved prediction of PCa and HGPCa. Adding WHR to a multivariable model increased the diagnostic accuracy for detecting HGPCa. Patients who are suspected of having PCa may benefit from WHR measurement.

## Methods

### Patients and variables

Consecutive patients were prospectively recruited from men undergoing prostate needle biopsy at the Shanghai Cancer Center, a tertiary hospital in China between March 2013 and October 2015. Indications for prostate biopsy were: A. PSA > 4.0 ng/ml; B. positive findings from digital rectal exam (DRE), with any level of PSA; or C. positive findings from imaging techniques, namely transrectal ultrasound (TRUS) or magnetic resonance imaging (MRI), with any level of PSA.

Before prostate biopsy, a questionnaire was used to retrieve every patient’s clinical information as life style (smoking history, drinking history) and comorbidities (hypertension, diabetes mellitus (DM)). In response to the question, “Have you ever smoked at least one cigarette a day for one year or longer”, subjects who answered “no” were classified as “never smokers”, those who answered “yes” were classified as “ever smokers”. Similarly, In response to the question, “Have you ever drunk at least 12.5 g alcohol a day for one year or longer”, subjects who answered “no” were classified as “never drinkers”, those who answered “yes” were classified as “ever drinkers”. Hypertension is defined as a systolic blood pressure above 140 mmHg or a diastolic blood pressure above 90 mmHg for more than one year. Pre-existing diabetes is defined as having a diagnosis of diabetes for more than one year before the prostate cancer was diagnosed. The definition of diabetes is: fasting blood glucose was greater than or equal to 7 mmol/L, and/or postprandial blood glucose was greater than or equal to 11.1 mmol/L. A professional research nurse then measured subjects’ height, weight, and waist and hip circumference and collected blood samples. All men underwent transrectal ultrasound-guided needle prostate biopsy with at least 10 cores. Primary outcomes were the pathological results of biopsy specimens. HGPCa was defined as the presence of a Gleason score ≥8. Pathological slides were reviewed by dedicated genitourinary pathologists in Shanghai Cancer Center. The study was carried out in accordance with the ethical standards of the Helsinki Declaration II and approved by the Institution Review Board of Fudan University Shanghai Cancer Center. Written informed consent was obtained from each patient before any study-specific investigation was performed.

### Statistical Analysis

**Figure 2:**
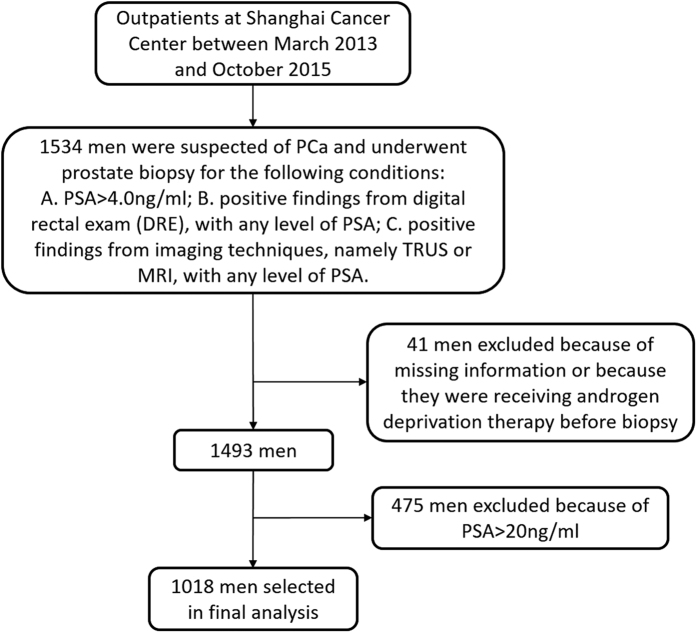
Flow diagram for entry and exclusion criteria.

Of 1,534 men that underwent prostate biopsy from March 2013 to October 2015 in Shanghai Cancer Center, 41 men were excluded because of missing information or because they were receiving androgen deprivation therapy before biopsy. Of the remaining 1,493 men, 1,018 men had PSA ≤20 ng/ml and were selected for further analyses in this study (Fig. 2). The rationale for limiting the analysis to men with PSA ≤20 ng/ml is that they are most likely to be over diagnosed^34^ and thus they require better screening tools to avoid unnecessary biopsy. Advanced PCa patients tended to have high level PSA (often >20 ng/ml) and cancer cachexia may cause unnecessary bias in our body measurement analyses.

Differences in patient characteristics were compared using the Kruskal-Wallis test for continuous variables, and the chi-squared test for categorical variables. Single variable and multivariable logistic regression analyses were used to generate ORs of BMI & WHR for detecting PCa and HGPCa at biopsy and for building prognostic models. Because of the small range of WHR (less than 0.5), we used WHR×100 instead in the regression models, which makes the OR and 95%Cl easier to interpret. We then used logistic regression to construct a multivariable model to predict PCa risk. Model 1 based on PSA, DRE results, and imaging (TURS or MRI) findings, which represented the various indications for biopsy we used in our population. Later, BMI and WHR were successively added to build new models. Area under curve (AUC) was used to measure discriminative ability. The improvement in AUC was tested using Delong·Clarke-Pearson test. Statistical analyses were performed using SPSS 20.0 and Stata 12.0. Significance was two-sided and set at P < 0.05.

## Additional Information

**How to cite this article:** Tang, B. *et al*. Waist-hip Ratio (WHR), a Better Predictor for Prostate Cancer than Body Mass Index (BMI): Results from a Chinese Hospital-based Biopsy Cohort. *Sci. Rep.* **7**, 43551; doi: 10.1038/srep43551 (2017).

**Publisher's note:** Springer Nature remains neutral with regard to jurisdictional claims in published maps and institutional affiliations.

## Supplementary Material

Supplementary Information

## Figures and Tables

**Table 1:** Clinical characteristics and biopsy outcomes of the study population stratified by biopsy outcome.

Variables	Total	Non-Cancer	Cancer
No.Patients	1018	763	255
Age, years	66 (60, 72)	65 (59, 70)	68 (63, 74)
PSA, ng/ml	9 (6, 12)	8 (6, 11)	10 (7, 14)
%fPSA	13.4 (9.2, 19.4)	14.9 (9.7, 20.9)	10.9 (8.4, 15.7)
Prostate volume, cm^3^	40 (30, 55)	45 (31, 60)	35 (24, 43)
DRE Abnormal (%)	9	8	16
TRUS or MRI Abnormal (%)	25	22	36
Height, cm	170 (166, 173)	170 (166, 173)	170 (165, 174)
Weight, kg	68 (61, 75)	68 (62, 75)	68 (60, 75)
Recent BMI, kg/m^2^	24 (22, 25)	24 (22, 25)	24 (22, 25)
Waist circumference, cm	86 (81, 91)	85 (81, 90)	86 (81, 91)
Hip circumference, cm	95 (91, 99)	95 (91, 99)	95 (90, 100)
WHR	0.903 (0.878, 0.929)	0.900 (0.876, 0.927)	0.907 (0.888, 0.934)
Smokers (%)			
ever	43	41	47
never	57	59	53
Alcohol use (%)			
ever	39	39	39
never	61	61	61
Hypertension (%)	34	33	36
Diabetes mellitus (%)	10	10	10
Biopsy outcome = cancer, n (%)	255 (25)	0	255 (100)
Gleason score, n (%)			
6	55 (5)	—	55 (22)
7	97 (10)	—	97 (38)
8	43 (4)	—	43 (17)
9	49 (5)	—	49 (19)
10	11 (1)	—	11 (4)

^^^Continuous variables are shown as the median value and interquartile range.

**Table 2:** Clinical characteristics and biopsy outcomes of the study population stratified by biopsy outcome detail.

Variables	Non-Cancer	Low grade Cancer (Gleason score = 6 or 7)	High grade Cancer (Gleason score >=8)	P-value
No.Patients	763	152	103	—
Age, years	65 (59, 70)	68 (62, 74)	68 (63, 75)	<0.001*
PSA, ng/ml	8 (6, 11)	10 (7, 13)	11 (8, 14)	<0.001*
%fPSA	14.9 (9.7, 20.9)	12.3 (8.7, 16.5)	10.8 (8.1, 15.1)	<0.001*
Prostate volume, cm^3^	45 (31, 60)	36 (29, 45)	33 (23, 42)	<0.001*
DRE Abnormal (%)	8	11	25	0.009**
TRUS or MRI Abnormal (%)	22	33	41	0.021**
Height, cm	170 (166, 173)	170 (165, 174)	170 (165, 173)	0.776*
Weight, kg	68 (62, 75)	68 (61, 75)	68 (60, 75)	0.618*
Recent BMI, kg/m^2^	24 (22, 25)	24 (22, 25)	24 (21, 25)	0.924*
Waist circumference, cm	85 (81, 90)	85 (80, 91)	87 (81, 91)	0.394*
Hip circumference, cm	95 (91, 99)	95 (90, 100)	95 (90, 99)	0.573*
WHR	0.900 (0.876, 0.927)	0.902 (0.882, 0.924)	0.918 (0.891, 0.938)	<0.001*
Smokers (%)				0.214**
ever	41	44	50	
never	59	56	50	
Alcohol use (%)				0.712**
ever	39	41	36	
never	61	59	64	
Hypertension (%)	33	35	38	0.651**
Diabetes mellitus (%)	10	10	9	0.924**
Biopsy outcome = cancer, n	0	152	103	—
Gleason score, n (%)				—
6	—	55 (36)	—	
7	—	97 (64)	—	
8	—	—	43 (42)	
9	—	—	49 (48)	
10	—	—	11 (10)	

^^^Continuous variables are shown as the median value and interquartile range.

^*^Using the Kruskal-Wallis test.

^**^Using the chi-squared test.

**Table 3:** OR, 95%Cl & P-value at Logistic regression of BMI & 100 × WHR in PCa and HGPCa detection.

Variables	Predictors of Cancer	Predictors of High-grade Cancer (Gleason >=8)		
	OR (95% CI)	p-value	OR (95% CI)	p-value
BMI				
Univariable	0.99 (0.94, 1.05)	0.799	0.97 (0.90, 1.05)	0.483
Multivariable*	1.02 (0.97, 1.07)	0.473	1.00 (0.92, 1.08)	0.932
WHR				
Univariable	1.06 (1.02, 1.09)	0.002	1.12 (1.06, 1.18)	<0.001
Multivariable*	1.07 (1.03, 1.11)	0.001	1.14 (1.09, 1.19)	<0.001

^*^Results adjusted for age, PSA, smoking status, alcohol use, hypertension and DM.

**Table 4:** OR, 95%Cl & P-value at multivariate Logistic regression* of BMI in PCa and HGPCa detection stratified by BMI group.

Variables	No.Patients	Predictors of Cancer		Predictors of High-grade Cancer (Gleason >=8)	
		OR (95% CI)	p-value	OR (95% CI)	p-value
Total	1018	1.02 (0.97, 1.07)	0.473	1.00 (0.92, 1.08)	0.932
Under and normal weight (BMI <22.9 kg/m^2^)	400	0.90 (0.77, 1.05)	0.191	0.92 (0.74, 1.15)	0.469
Overweight (BMI 23–24.9 kg/m^2^)	330	1.13 (0.73, 1.78)	0.577	1.28 (0.67, 2.43)	0.463
Moderately obese (BMI 25–29.9 kg/m^2^)	272	0.89 (0.68, 1.15)	0.378	0.80 (0.55, 1.16)	0.247
Severally obese (BMI ≥ 30 kg/m^2^)	16	/**	/	/**	/

^*^Results adjusted for age, PSA, smoking status, alcohol use, hypertension and DM.

^**^Too small sample size to do logistic regression.

**Table 5:** AUC, 95%Cl & P-value in different screening models.

Model	Predictors of Cancer		Predictors of High-grade Cancer (Gleason >= 8)	
	AUC	p-value*	AUC	p-value*
Model 1**	0.645 (0.605, 0.684)	reference	0.660 (0.602, 0.717)	reference
Model 1 + BMI	0.645 (0.606, 0.685)	0.508	0.661 (0.603, 0.718)	0.718
Model 1 + WHR	0.655 (0.617, 0.693)	0.245	0.706 (0.653, 0.758)	0.025

^*^Using the Delong·Clarke-Pearson test to compare AUC of the ROC curves.

^**^Model 1: PSA + DRE + imaging techniques (TRUS or MRI).
