# Supplementary material for: Waist-hip Ratio (WHR), a Better Predictor for Prostate Cancer than Body Mass Index (BMI): Results from a Chinese Hospital-based Biopsy Cohort
Source: Sci Rep. 2017 Mar 8;7:43551. doi: 10.1038/srep43551 (PMC5341100; doi:10.1038/srep43551)
Supplement: Supplementary Information [file srep43551-s1.pdf]

Supplementary Information

Waist-hip Ratio (WHR), a Better Predictor for Prostate Cancer than Body Mass Index (BMI): Results from a Chinese Hospital-based Biopsy Cohort

Bo Tang[1,2], Cheng-Tao Han[1,2], Gui-Ming Zhang[1,2], Cui-Zhu Zhang[1,2], Wei-Yi Yang[1,2], Ying Shen[1,2], Adriana C. Vidal[3], Stephen J. Freedland[3], Yao Zhu[1,2], Ding-Wei Ye[1,2]

[1] Department of Urology, Fudan University Shanghai Cancer Center, Shanghai, China
[2] Department of Oncology, Shanghai Medical Colleague, Fudan University, Shanghai, China
[3] Department of Surgery, Center for Integrated Research on Cancer and Lifestyle, Samuel Oschin Comprehensive Cancer Institute, Cedars Sinai Medical Center, Los Angeles, CA
Correspondence and requests for materials should be addressed to Yao Zhu (e-mail: mailzhuyao@gmail.com) or Ding-Wei Ye (e-mail:yedingwei1963@126.com)

Supplementary Fig 1

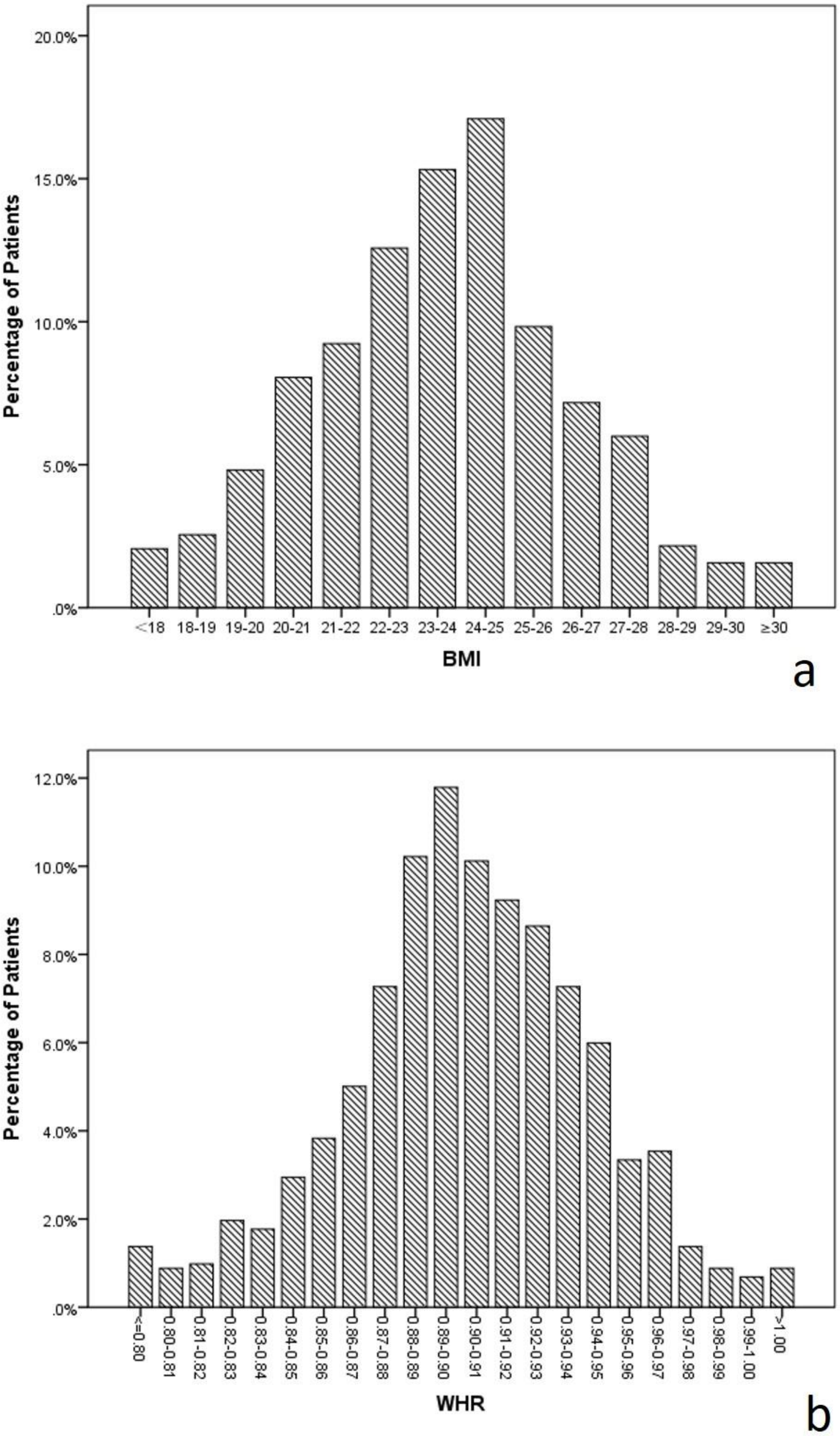

Supplementary Fig 1a. Distribution of BMI in the study population.

Supplementary Fig 1b. Distribution of WHR in the study population.
